# Effect of the Transradial Approach on Wrist Function in Diagnostic Cerebral Angiography

**DOI:** 10.3390/healthcare14020254

**Published:** 2026-01-20

**Authors:** Julian Kifmann, Michael Braun, Johannes Steinhart, Nico Sollmann, Christopher Kloth, Maria Pedro, Jens Dreyhaupt, Meinrad Beer, Bernd Schmitz, Johannes Rosskopf

**Affiliations:** 1 Section of Neuroradiology, Bezirkskrankenhaus Guenzburg, 89312 Guenzburg, Germany; 2 Department of Oral and Plastic Maxillofacial Surgery, Armed Forces Hospital Ulm, 89081 Ulm, Germany; 3 Department of Diagnostic and Interventional Radiology, University Hospital Ulm, 89081 Ulm, Germany; 4 Department of Nuclear Medicine, University Hospital Ulm, 89081 Ulm, Germany; 5 Department of Diagnostic and Interventional Neuroradiology, School of Medicine and Health, TUM Klinikum Rechts der Isar, Technical University of Munich, 81675 Munich, Germany; 6 TUM-Neuroimaging Center, TUM Klinikum Rechts der Isar, Technical University of Munich, 81675 Munich, Germany; 7 Section of Peripheral Nerve Unit, Bezirkskrankenhaus Guenzburg, 89312 Guenzburg, Germany; 8 Department of Neurosurgery, Bezirkskrankenhaus Guenzburg, 89312 Guenzburg, Germany; 9 Institute of Epidemiology and Medical Biometry, University of Ulm, 89075 Ulm, Germany

**Keywords:** neurointervention, transradial access, diagnostic cerebral angiography

## Abstract

**Background**: With increasing demand for ambulatory catheter angiography, interest in the transradial approach for diagnostic cerebral procedures has grown markedly. This study aimed to evaluate the effect of the transradial approach for catheter-based diagnostic cerebral procedures on wrist function. **Methods**: Wrist function was quantified by the Patient-Rated Wrist Evaluation (PRWE) questionnaire. The PRWE score ranged from 0 to 100, with 0 indicating no functional impairment. Association analyses with demographic and clinical parameters were performed using univariate logistic regression models. **Results**: A total of 88 patients underwent ambulatory diagnostic cerebral angiography during the 15-month observation period; of these, 40 (45%) participated in a telephone interview. Overall, 47.5% (*n* = 19) of patients reported no wrist impairment (PRWE = 0) after the transradial approach. The remaining 52.5% (*n*= 21) showed a mean PRWE score of 21.3 ± 22.5 (standard deviation), with a median value of 11.0 and a range from 1.0 to 87.0. Interestingly, univariate logistic regression models revealed a trend towards association between the dichotomized PRWE score and body mass index (*p* = 0.051). No associations were found with age, sex, prior neurosurgical status, total procedure duration, dose area product, fluoroscopy time, or dominant hand (*p* > 0.05). **Conclusions**: Following transradial cerebral catheter angiography, 52.5% of patients reported some degree of wrist impairment at follow-up; whether this represents procedure-related deterioration cannot be determined without baseline values.

## 1. Introduction

Diagnostic cerebral catheter angiography remains an essential modality for a wide range of neurovascular disorders, in spite of major advances in non-invasive imaging. It continues to be the reference standard for many indications, particularly for the peri-procedural evaluation of endovascularly treated aneurysms, intracranial stenoses, and dural arteriovenous fistulas.

Cerebral catheter angiography has evolved from early direct puncture techniques used for traumatic carotid–cavernous fistulas into a modern modality that allows detailed imaging of intracranial vascular pathology and facilitates advanced intravascular treatments [1]. In neuroradiology, the transfemoral approach has traditionally been the standard for diagnostic catheter angiography. Therefore, patients are usually observed for at least one night to monitor for puncture-site bleeding and cerebral ischemia [2,3]. In interventional cardiology, the radial-artery-first strategy has become standard due to lower bleeding rates, fewer vascular complications, and improved patient comfort, leading to shorter hospital stays [4,5,6,7]. These advantages have driven increasing adoption of transradial access for diagnostic cerebral angiography, particularly in outpatient settings [8].

Despite its growing use, transradial access is associated with specific challenges, including longer procedure times, anatomical limitations, and the potential need for crossover to transfemoral access [9,10,11]. Access-site complications can also result in impaired wrist function [12,13]. The underlying mechanisms remain poorly understood. Radial artery occlusion is among the most common complications of the transradial approach. Acute ischemia is rare due to the dual arterial supply of the forearm [14]. Intimal thickening, endothelial dysfunction, and nerve damage may also contribute to the development of wrist dysfunction [15,16]. However, to date, only a few studies in cardiology have investigated upper limb dysfunction after transradial access [12,13,17]. It is assumed that such complications may be underreported, either due to insufficient recognition during follow-up examinations or a reluctance among operators to acknowledge them [12,18]. However, wrist function is essential for many activities of daily living [19,20].

Wrist function assessment is challenging, as reliable measurement is required to identify potential functional impairment [21,22]. To date, clinical assessment scales and questionnaires remain the most commonly used tools for evaluating wrist function [23,24]. Although various objective measurement techniques have been described, no single standardized and universally applicable method for instrumental wrist function assessment is currently available [25]. The most established scoring system, which is used in the current study, is the Patient-Rated Wrist Evaluation (PRWE) questionnaire [19,20].

Against this background, the present study aimed to investigate the effects of the transradial access on wrist function for diagnostic cerebral procedures. Wrist function was assessed using the established self-reported Patient-Rated Wrist Evaluation (PRWE) questionnaire [19,20].

## 2. Materials and Methods

### 2.1. Study Design and Patient Selection

The transradial approach is the primary access route for diagnostic cerebral catheter angiography at our institution. All patients who underwent diagnostic transradial angiography between January 2020 and March 2021 were retrospectively identified from our institutional database. According to the direction of the local ethics committee, written informed consent from each patient had to be achieved before contacting via telephone. Following data collection, all patient information was fully anonymized prior to analysis. The study was approved by the local ethics committee (reference number 238/21). The study cohort has previously been reported [26]. However, no investigations of wrist function have previously been reported.

### 2.2. Transradial Approach

Patients were investigated in the supine position. Using an angiography arm board, the forearm was secured to allow access the radial artery. Under sterile conditions, approximately 10 mL of local anesthetic (Prilocain Hydrochlorid 10 mg/mL; Xylonest^®^ 1%, Aspen Germany GmbH, Munich, Germany) was administered subcutaneously. The local anesthetic served to minimize both procedural pain and vasospasm. No preprocedural testing of collateral hand circulation (e.g., an Allen’s or Barbeau test) was performed. Ultrasound guidance was not routinely used for vascular access.

Radial artery access was achieved by palpation using a 20-gauge puncture needle, followed by insertion of a 5-French access device (Glidesheath Slender^®^; hydrophilic coated introducer sheath, Terumo Germany GmBH, Eschborn, Germany) using the Seldinger technique (with a Flex Straight^®^ 0.021″ × 45 cm mini guidewire and an 0.021″ dilator; Terumo Germany GmBH, Eschborn, Germany). To prevent vasospasm and thrombotic events, intra-arterial administration of verapamil (2.5 mg) and heparin (5000 U) was performed slowly over 2–3 min to reduce the risk of hypotension.

To confirm correct intravascular sheath placement and rule out vascular injury or anatomical anomalies, radial artery angiography was performed. The sheath was not connected to a continuous flush system. Selective catheterization of target vessels was conducted using a 0.035′′ guidewire (Terumo Germany GmBH, Eschborn, Germany or Asahi Intecc Europe B.V., Frankfurt, Germany) in combination with a diagnostic catheter, in most cases a 5-French Simmons-2 catheter (Penumbra Neuron Select^®^, Berlin, Germany). The diagnostic catheter was continuously flushed with heparinized saline solution throughout the procedures.

At the end of the angiography, hemostasis was achieved using a TR BAND^®^ radial compression device (Terumo Germany GmBH, Eschborn, Germany), allowing air titration to ensure patent hemostasis. The compression band was gradually deflated over a period of two hours and then removed. Patients remained mobile during this period.

### 2.3. Telephone Interview

All patients were contacted by mail in August 2021 regarding consent for a telephone interview. The purpose of the telephone interview was to assess wrist function after angiography using the established self-reported PRWE questionnaire [19,20,27]. Introduced by MacDermid in 1996 and updated in 2019 [19,27], the PRWE is a 15-item questionnaire designed to measure wrist pain and disability in activities of daily living. The score ranges from 0 to 100, with 0 indicating no functional impairment.

In 2018, Mulders et al. provided a representative overview of the normative data for the normal population [20]. Briefly, in the Netherlands, visitors and employees of four hospitals were asked to participate. A total of 1042 eligible participants were approached for analysis, and normative data for the PRWE score were reported as a mean ± standard deviation (SD) of 7.7 ± 15, with a median of 0 and interquartile range from 0 to 8.5. The median age of participants was 51 years, and 44% were men.

### 2.4. Statistical Analysis

All statistical analyses were performed using IBM SPSS Statistics (Version 30; IBM Corp., Armonk, NY, USA) and SAS, version 9.4 (The SAS Institute, Cary, NC, USA).

Demographic data and other collected variables were reported with descriptive statistics. PRWE scores and continuous variables were presented as means with corresponding standard deviations (SDs), as well as minimum, maximum, and median values. For PRWE scores the interquartile range was also reported. Categorical variables were reported as absolute numbers and percentages.

The study cohort was stratified into participants with (Group A) or without (Group B) wrist impairment, as defined by a PRWE score greater than zero or equal to zero, respectively. As the PRWE data showed a marked deviation from a normal distribution the outcome was dichotomized. Univariate logistic regression models were then used to explore potential associations with patients’ characteristics and procedural variables, allowing the results to be reported as odds ratios (ORs). Because of model convergence issues, the dose area product (DAP) was categorized into two groups based on its median value. A two-sided *p*-value < 0.05 was considered statistically significant. Due to the explorative nature of this study and the aforementioned considerations, no adjustment for multiple testing was made.

The purpose of the univariate logistic regression model was to evaluate the individual association between each potential predictor and the binary outcome (PRWE > 0 vs. PRWE = 0), in order to identify whether any variable showed a meaningful effect that would justify further multivariable modeling. As no relevant associations were observed and the sample size was limited, a multivariable model was neither meaningful nor statistically robust.

## 3. Results

### 3.1. Study Sample

Between January 2020 and March 2021, a total of 88 patients were enrolled. Overall, 40 patients (45%) participated in the telephone interview, with mean follow-up time being 10.3 months (median: 10.0 months). The mean age of the study sample was 57.1 ± 12.5 years (median of 57.0 years; min: [range: 25.0 years–81.0 years]). Overall, 62.5% of were female.

Cerebral catheter angiography was performed in 80% of cases following surgery, and in 85% of cases with radial access on the right side. In 80% of cases, vascular access was obtained via the dominant hand. Access site complications (vasospasm) occurred in five cases (12.5%). Further patient characteristics and procedural variables stratified into participants with (Group A) or without (Group B) wrist impairment are summarized in Table 1.

### 3.2. Wrist Function

**Figure 1:**
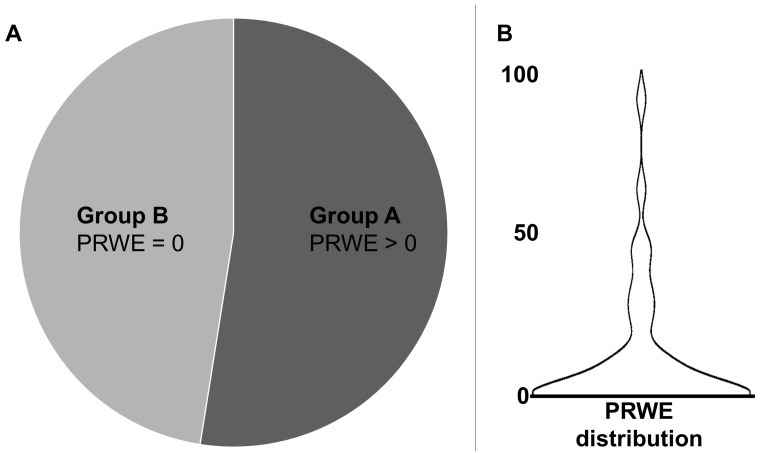
Based on the Patient-Rated Wrist Evaluation (PRWE) questionnaire, 47.5% of patients (*n* = 19, Group B) reported no wrist impairment (PRWE = 0), while 52.5% (*n* = 21, Group A) had a PRWE > 0 (**A**). The violin plot shows the PRWE distribution (**B**) including all patients (*n* = 40).

Using PRWE, 47.5% of patients (*n* = 19) did not complain about any wrist impairment after transradial access (PRWE = 0; Group B). The remaining 52.5% (21 patients, Group A) showed a mean PRWE of 21.3 ± 22.5 (standard deviation). The median was 11.0, with a minimum–maximum range of 1.0–87.0 and an interquartile range of 6.75–30.25. The entire study sample (*n* = 40) had a median PRWE score of 1.0, with an interquartile range of 0–11.38 and a mean of 11.18 (Figure 1).

Compared to normative data, 47.5% and 62.5% of patients in the overall cohort had PRWE scores at or below the normative median of 0 and the normative mean of 7.7, respectively [20]. Thirty-two patients (80%) scored within one standard deviation of the normative data (mean + SD = 22.7).

### 3.3. Association Analyses

In the investigated cohort, univariate logistic regression models revealed a trend towards association between the dichotomized PRWE score and BMI; that is, a higher PRWE score was associated with a higher BMI (*p* = 0.051). No further associations were found with age, sex, prior neurosurgical status, total procedure duration, DAP, fluoroscopy time, vasospasm, or dominant hand (*p* > 0.05 each, Table 2).

In a sensitivity analysis excluding patients with a follow-up longer than 12 months, the associations remained non-significant in the regression models (*p* > 0.05 each).

## 4. Discussion

In this study, wrist function following a transradial approach for diagnostic cerebral catheter angiography was evaluated using the established patient-reported PRWE questionnaire. Overall, 47.5% of patients reported no wrist impairment after the transradial approach in the retrospective study design. Interestingly, univariate logistic regression models revealed a trend toward a significant association between the dichotomized PRWE score and body mass index (*p* = 0.051). No further associations were found with age, sex, prior neurosurgical status, total procedure duration, DAP, fluoroscopy time, or vasospasms.

In general, the PRWE questionnaire was specifically developed to assess wrist-related function and pain [27], thus offering high specificity and sensitivity, and aligning well with our clinical observations that the transradial approach primarily affects the wrist. In contrast, the Quick-DASH questionnaire [28], a shortened version of the Disabilities of the Arm, Shoulder, and Hand (DASH) questionnaire that evaluates the entire upper extremity and is commonly used in cardiology studies, was deemed less suitable for this purpose.

In the current study, 47.5% of patients reported no wrist impairment (PRWE = 0) after transradial access. Accordingly, 52.5% had PRWE scores above zero, suggesting that some degree of wrist dysfunction was present in about half of the patients. Therefore, wrist impairment may occur after transradial access, and this should be considered when evaluating post-procedural outcomes.

Granted, a causal relationship between transradial access and higher PRWE scores should be interpreted with caution, as other factors may also contribute. Thus, compared to normative data, in the current study 80% of patients scored within one standard deviation of the normative data for PRWE (mean + SD = 22.7) [14]. Moreover, according to Walenkamp et al. [29], the minimum clinically important difference for the PRWE is 11.5 points, a finding based on a prospective cohort of 102 patients with distal radius fractures undergoing upper extremity surgery. In the present study, the observed wrist impairment might not reflect a clinically meaningful limitation for most patients, given that 77.5% had PRWE scores between zero and 11.5 points.

Moreover, our findings are consistent with previous prospective studies in interventional cardiology that evaluated the impact of transradial coronary catheterization on upper limb function at follow-up intervals of two weeks and thirty days [12,17]. Zwaan et al. found wrist dysfunction at two-week follow-up examinations, and reported that upper extremity impairment occurred twice as often in the transradial group compared to the transfemoral group (*n* = 440 vs. 62) [12]. These early upper extremity complications have been shown to resolve within six months. Another interventional cardiology study [17] involving 338 patients found a decrease in mean Quick-DASH scores from 4.55 at baseline to 2.27 at 30-day follow-up examinations. As the change did not reach statistical significance (*p* = 0.06), the authors concluded that upper limb function was not significantly affected by transradial coronary catheterization, and this finding was confirmed in a follow-up study involving long-term evaluation with a one-year follow-up interval [13]. In two systematic reviews [30,31], hand dysfunction following cardiac catheterization was found to be rare (0.16% to 7.77%). It was noted in these reviews that patients may experience non-specific sensory and motor complaints, which typically resolve over time.

In the present study, association analysis revealed a trend towards an association between the dichotomized PRWE score and BMI (*p* = 0.051). In the light of the multiple comparisons and small sample size, this trend should be regarded as exploratory in nature and be interpreted as hypothesis-generating rather than indicative of a true relationship. Potential underlying mechanisms may include increased difficulty in tissue dissection and differences in inflammatory response. Moreover, late-onset symptoms are unlikely, as most TRA complications occur early. In a sensitivity analysis excluding patients with a follow-up longer than 12 months, the associations remained non-significant in the regression models (*p* > 0.05 for each). Nevertheless, the median follow-up of approximately 10 months in this study is relatively long, and this should be taken into account when interpreting the findings. For plausibility, PRWE scores are known to vary by sex and were, as expected, in our study higher in women than in men.

In summary, the clinical significance of the present findings is that patients should be adequately informed about potential wrist deterioration. Wrist impairment is minor for most individuals, while particular attention should be paid to the minority experiencing severe symptoms. Persistent complaints should prompt clinical reassessment.

A limitation of this study was the retrospective design which precluded the collection of baseline PRWE scores prior to the procedure. However, given that the aim of the study was to provide a general assessment of the impact of the elective transradial access for diagnostic cerebral angiography on wrist function, the comparison with established normative data from the validated PRWE questionnaire was deemed sufficient. Additionally, the cohort in the current study included slightly older patients and a higher proportion of women compared to the Dutch population used for the normative data which are known to be associated with higher PRWE scores independent of any procedure. Furthermore, the response rate of 45% for the telephone survey seems to be average, yet underscores that not all consecutive patients were assessed. The lack of information on non-responders limits the ability to assess potential response bias. It remains highly speculative but at least possible that symptomatic individuals were either more motivated or, conversely, less reachable, thereby biasing the estimates in either direction. Moreover, even though we monitored the distribution of participants regarding age and sex, type of employment and smoking status were not assessed, even though these factors are known to influence PRWE scores [20]. Future prospective studies on the transradial approach may address these limitations, and thus improve risk stratification related to wrist function.

## 5. Conclusions

More than half of patients reported some wrist symptoms after transradial diagnostic cerebral angiography. Because of the retrospective study design, no baseline measurements were available. Therefore, while wrist symptoms were reported, causality cannot be established with regard to whether they represented a procedure-related deterioration. Nevertheless, clinical relevance does appear limited in most cases. These findings underscore the importance of long-term functional follow-up in future prospective studies of transradial neurointerventional procedures.

## Figures and Tables

**Table 1:** Patients’ characteristics and procedural variables.

Variable	Group A (*n* = 21) ^1^	Group B (*n* = 19) ^2^
Age (years) ^3^	59.9 ± 11.3; 58.0 [38.0–81.0]	54.1 ± 13.4; 57.0 [25.0–75.0]
Sex (m/f) ^4^	8/13	7/12
Angiography before/after ^5^ endovascular treatment	3/18	5/14
BMI (kg/m^2^) ^6^	28.9 ± 7.8; 28.1 [17.0–54.0]	24.8 ± 3.3; 25.1 [19.6–31.7]
DAP (µGym^2^) ^7^	5010.2 ± 5022.5; 3884.8 [1025.5–25,557.0]	5139.8 ± 4599.8; 3748.0 [1077.9–17,346.0]
Total duration (minutes) ^8^	21.6 ± 9.8; 18.0 [6.0–42.0]	24.6 ± 18.8; 20.0 [7.0–86.0]
Fluoroscopy time (minutes) ^9^	9.2 ± 5.6; 9.1 [3.0–27.8]	12.1 ± 10.9; 8.7 [2.1–42.0]
Vasospasm (yes/no) ^10^	3/18	2/17

^1^ Group A consisted of the 21 patients with a deterioration in wrist function, indicated by a PRWE score of >0; ^2^ Group B comprised all other cases. ^3^ Mean ± standard deviation; median [min–max]. ^4^ male/female. ^5^ Number of patients before/after endovascular treatment. ^6^ Body mass index. ^7^ Dose area product. ^8^ Total duration of the procedure in the angiosuite. ^9^ Fluoroscopy time of the procedure in the angiosuite. ^10^ Number of patients with vasospasm versus no vasospasm (yes/no).

**Table 2:** Results of the association analysis using univariate logistic regression models.

Variable	Odds Ratio ^1^	95%-CI ^2^	*p*-Value ^3^
Age (years)	1.041	0.986–1.100	*p* = 0.148
Sex (m/f) ^4^	1.055	0.293–3.803	*p* = 0.935
Angiography before/after endovascular treatment	1.417	0.210–9.548	*p* = 0.721
BMI (kg/m^2^) ^5^	1.170	1.000–1.370	***p*** **=** **0.051**
DAP (µGym^2^) ^6^	1.222	0.353–4.235	*p* = 0.752
Total duration (minutes) ^7^	0.985	0.942–1.030	*p* = 0.518
Fluoroscopy time (minutes) ^8^	0.958	0.884–1.039	*p* = 0.301
Hand dominance ^9^	0.874	0.241–5.341	*p* = 1.134

Wrist impairment was the dependent variable (PRWE > 0 vs. PRWE = 0). Univariate logistic regression models showed a trend toward association between PRWE (Patient-Rated Wrist Evaluation) and BMI (body mass index); the corresponding *p*-value was highlighted in bold. ^1^ Values represent odds ratios with ^2^ 95% confidence intervals (CI) and ^3^ *p*-values. ^4^ Male/female. ^5^ Body mass index. ^6^ Dose area product, categorized on its median value due to the model convergence issue. ^7^ Total duration of the procedure in the angio-suite. ^8^ Fluoroscopy time of the procedure in the angiosuite. ^9^ Vascular access was obtained via the dominant hand (or not).

## Data Availability

The data that support the findings of this study are available from the corresponding author upon reasonable request. Due to ethical and legal restrictions related to the protection of participant privacy and confidentiality, the raw data cannot be made publicly available.

## Abbreviations

The following abbreviations are used in this manuscript:

DAP	Dose Area Product
DASH	Disabilities of the Arm, Shoulder, and Hand
PRWE	Patient-Rated Wrist Evaluation
Quick-DASH	Shortened version of Disabilities of the Arm, Shoulder, and Hand
